# Microfluidics on lensless, semiconductor optical image sensors: challenges and opportunities for democratization of biosensing at the micro-and nano-scale

**DOI:** 10.1515/nanoph-2023-0301

**Published:** 2023-10-13

**Authors:** Xinyue Hu, Reza Abbasi, Sebastian Wachsmann-Hogiu

**Affiliations:** Department of Bioengineering, McGill University, Montreal, QC H3A 0C3, Canada

**Keywords:** optical image sensors, lensless imaging, microfluidics, device integration, point of need

## Abstract

Optical image sensors are 2D arrays of pixels that integrate semiconductor photodiodes and field effect transistors for efficient photon conversion and processing of generated electrons. With technological advancements and subsequent democratization of these sensors, opportunities for integration with microfluidics devices are currently explored. 2D pixel arrays of such optical image sensors can reach dimensions larger than one centimeter with a sub-micrometer pixel size, for high spatial resolution lensless imaging with large field of view, a feat that cannot be achieved with lens-based optical microscopy. Moreover, with advancements in fabrication processes, the field of microfluidics has evolved to develop microfluidic devices with an overall size below one centimeter and individual components of sub-micrometer size, such that they can now be implemented onto optical image sensors. The convergence of these fields is discussed in this article, where we review fundamental principles, opportunities, challenges, and outlook for integration, with focus on contact-mode imaging configuration. Most recent developments and applications of microfluidic lensless contact-based imaging to the field of biosensors, in particular those related to the potential for point of need applications, are also discussed.

## 1 Introduction

Semiconductors are materials used to build main components of modern electronics ranging from smartphones to solar cells [1]. One of the main components of these electronics is the field-effect transistor that utilizes n- and p-doped semiconductor materials for electronic switches and amplifiers. These transistors can be manufactured with different architectures, with one of the most widely used being the complementary metal-oxide-semiconductor (CMOS) transistor consisting of a pair of p-type metal-oxide-semiconductor (PMOS) transistor and n-type metal-oxide-semiconductor (NMOS) transistor. The PMOS transistor has low resistance between its source and drain when a low voltage is applied, whereas NMOS transistor has low resistance under high applied voltage. By connecting the sources and drains of PMOS and NMOS transistors, CMOS transistors achieve reduced resistance regardless of the applied voltage. This reduced resistance leads to lower power consumption, reduced heat generation, and therefore lower noise.

The CMOS architecture has been implemented into image sensors, where a photodiode pixel is accompanied by CMOS transistors that amplify and digitize the electrons converted by individual semiconductor photodiodes. Since its initial appearance in 1967 [2], the development of these CMOS image sensors has progressed significantly. The conventional architecture of CMOS image sensors is planar, with the pixel array and readout circuits situated in the same plane [3]. In this configuration, the main building blocks of a sensor include a layer of microlenses deposited on top of the pixel array to focus the incoming light, a color filter array for color imaging, a pixel array that converts incoming photons into voltage signals, pre-amplifiers to enhance signal-to-noise ratio and dynamic range, analog-to-digital converters (ADCs) to convert the voltage signals into digital values, and digital I/O with on-chip memory and column decoder to store and output digitized results [3, 4]. By concentrating light to the photo-sensitive area, the layer of microlenses on top of the pixel array compensates for the loss of fill factor, which is the ratio of a pixel’s photo-sensitive area to the total area of each pixel. There are two main technologies for the planar CMOS image sensors described before: front-side illumination (FSI) where the photodiodes are placed below a metal stack of interconnects, and back-side illumination (BSI) where the photodiodes are placed above the metal stack of interconnects and are exposed first to the incident light [3]. In recent years, 3D-stacked BSI CMOS image sensor has been developed, where the pixel array and readout circuits are separated into different planes, giving rise to state-of-the-art commercial devices with a pixel count of 50 megapixels or higher, frame rate of 240 fps, and pixel size of 0.67 μm. A comprehensive comparison of these technologies is available in a review article [3].

An alternative to CMOS technology, charge-coupled devices (CCDs) were developed starting in 1970 [5], with both offering competing alternatives for various vision applications. While both CCD and CMOS image sensors are imaging sensors that convert incident light into digital values using photodetectors and readout circuits, the primary difference between the two arises from their readout mechanisms. CCDs perform the readout serially, with electric signals being transferred from pixel to pixel and then converted to digital values by an ADC [6]. In comparison, each pixel contains its own ADC in a CMOS image sensor with pixel-level readout architecture, enabling parallel readout and potentially faster readout speeds compared to a CCD [3]. Additionally, CMOS image sensor operates at significantly lower power consumption, typically 100 times lower than a CCD, with CMOS image sensor capable of operating in the milliwatt range and CCD operating in the watt range. On the other hand, the reduced in-pixel circuitry in CCD’s architecture leads to a higher fill factor, often enabling production of better image quality with lower noise. As CCD and CMOS image sensor technologies continue to evolve, the choice between CCD and CMOS image sensors depends on the specific application and the desired trade-off between readout speed, power consumption, image quality, and noise level.

In parallel to CMOS and CCD manufacturing processes, the microfluidics field has evolved since the 80s, to address problems related to handling small volumes of liquids (such as samples or reagents), finding a myriad of applications, from biomedical diagnosis to biosensors used in environmental monitoring, food safety, etc. Such characteristics drew significant attention to microfluidics systems for cases where samples are limited or difficult to collect, a growing challenge in biomedical applications [7]. In the following decades, transition from glass and silicon wafers to polymers and adaptation of biochemical reactions to microfluidics platforms, led to a wide use of these systems in molecular biology, biochemistry, and bioengineering, collectively referred to as bio-microfluidics [8]. Such systems proved to be excellent candidates to not only accommodate manipulation of biological samples in small volumes or at small length-scales but can also mimic *in-vivo* environment.

In a matter of years, and with the advent of lab-on-a-chip technologies, microfluidics can perform the whole cycle of sample-to-result and be used for diagnostics. Such point-of-need devices provide potentially inexpensive, disposable, and easy-to-use alternatives to centralized diagnosis facilities [9]. By integrating microfluidic devices onto an image sensor capable of capturing both the shadow cast by the sample and the optical signals resulting from analyte-specific reactions within the microfluidic component, a compact, standalone biosensing system can be achieved. This integrated system offers enhanced throughput and sensitivity, providing versatile applications ranging from cell-counting to analyte quantification across domains spanning from food safety to health monitoring. However, initial promises have been met with challenges, often specific to physical parameters of the system, such as choice of material, method of fabrication, and detection strategies.

This paper aims to provide a comprehensive overview of the integrated fields of microfluidics and lensless imaging, with a specific focus on contact-mode designs. We will start by discussing the fundamental principles, advantages, and applications of lensless imaging, highlighting its achievements as a cost-effective and high-throughput imaging technique. Next, we will review the principles and fabrication of microfluidic devices as well as the challenges involved in integrating them with lensless platforms. We will then introduce the advancements in the integrated fields of microfluidics on lensless image sensors and discuss the various requirements and approaches for creating such integrated platforms. Finally, we will conclude by offering an outlook on the future of this field, highlighting the potential for further innovation and applications in areas such as point-of-need diagnostics and health monitoring.

## 2 Lensless imaging

Bioimaging and biosensing focus on analyzing samples at the micrometer or nanometer level. Conventional bioimaging techniques use lenses to magnify the image and thereby increase the spatial resolution. With advancements in digital image sensors, lens-based digital imaging combines magnifying lenses with these sensors to rapidly acquire high-resolution images that are easily stored and processed. The combination of CMOS image sensor in mobile phones and external magnifying lenses marks the beginning of portable and accessible microscopic imaging technology [10–12]. However, the lenses introduce various limitations and drawbacks. First, lens-based imaging techniques face a challenge in balancing between spatial resolution and field of view (FOV) [13]. The FOV refers to the extent of the observable area captured by the sensor, and an increase in spatial resolution in lens-based imaging often results in a reduction of FOV [12]. Second, the lens introduces optical aberrations such as defocus and image distortion [10]. Finally, the images produced by lens-based imaging only show contrasts in intensity, which is not enough information for three-dimensional (3D) measurements. On the other hand, lensless imaging does not face restrictions imposed by the lenses and is therefore able to produce aberration-free high-resolution images without sacrificing the FOV. Specifically, the spatial resolution of lensless imaging is determined by factors including the pixel size of the image sensor and the SNR, while the FOV is equivalent to the active area of the image sensor which can reach 30 mm^2^ for CMOS image sensor and 20 mm^2^ for CCD [14], for pixels as small as 0.7 μm. To put it into perspective, a bench-top optical microscope with a 10 × objective lens with a typical numerical aperture of 0.2 has a FOV of less than 4 mm^2^ and a theoretical spatial resolution of 1.5 μm, which significantly limits the amount of sample per image [15]. Furthermore, lensless digital holographic imaging offers the added capability of depth-resolved 3D imaging. The elimination of lenses also provides additional benefits of enhanced portability and cost-effectiveness.

### 2.1 Main concepts in lensless imaging

**Figure 1:**
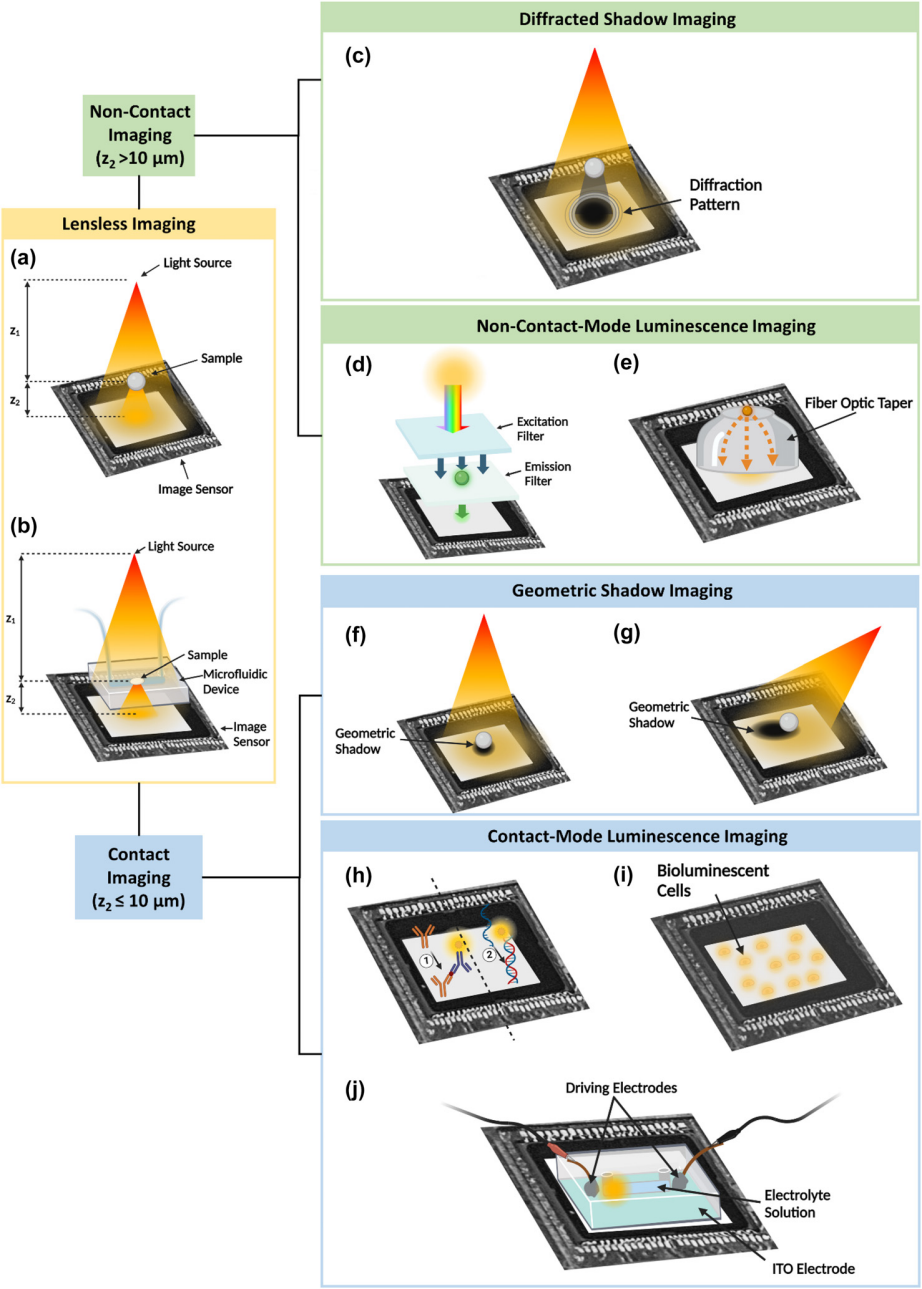
Methodologies for lensless on-chip imaging. (a) General schematic of lensless on-chip imaging. The light source can be coherent or partially coherent, with the aperture size adjusted to meet spatial coherence requirements. The light source-sample distance (*z*_1_) determines the illumination characteristics, and the sample-sensor distance (*z*_2_) is used to define contact or non-contact modes. (b) General schematic of lensless on-chip imaging with a microfluidic component. (c) General configuration for diffraction shadow imaging, where the sample is more than 10 μm from the image sensor surface and a diffraction pattern is captured by the image sensor. (d) Schematic of a fluorescence imaging platform. Conventional emission filters are thicker than 10 μm, exceeding the limit for contact-mode imaging. (e) Schematic of non-contact-mode luminescent imaging where a luminescent bead is connected to an image sensor through a fiber optic taper. (f) General setup of geometric shadow-based contact imaging, where the sample is directly placed on the image sensor and a geometric shadow with minimal diffraction is captured by the image sensor. (g) Configuration of oblique illumination in a geometric shadow-based contact imaging platform, where a point source LED provides illumination from different angles to create shadows of different lengths. (h) Demonstrations of chemiluminescence contact imaging with a luminophore-labeled sandwich immunoassay and a luminophore-labeled DNA hybridization assay. (i) General setup of bioluminescence contact imaging, where the bioluminescent cells are placed directly on the image sensor. (j) Schematic of a microfluidic integrated electrochemiluminescence contact imaging platform [17]. For luminescent samples described in (e, h, i, j), a light source is not required. *Modified with permission from* [17].

Lensless on-chip imaging techniques are a set of imaging methods that use a compact configuration without the need for bulky lenses, enabling high-resolution and wide field-of-view imaging. The basis of lensless on-chip imaging is the use of an image sensor, most commonly CMOS image sensor or CCD, to capture the diffraction patterns, shadow patterns, or luminescence emission generated by the object of interest [16]. In a lensless on-chip imaging platform, external lenses are absent, revealing the microarray of optical elements on the sensor surface. The general configuration of lensless imaging is illustrated in Figure 1a, where the light source is positioned at a distance *z*_1_ above the sample, and the sample is placed at a distance *z*_2_ above the surface of the image sensor. As shown in Figure 1b, lensless imaging can also be achieved with a microfluidic component for sample handling.

Different methodologies for lensless on-chip imaging and their classification are highlighted in Figure 1. Lensless imaging can be categorized as contact imaging and non-contact imaging based on the proximity between the sample and the sensor surface. In lensless contact imaging, the sample is in direct contact with the active area of the sensor, with *z*_2_ ≤ 10 μm, allowing for the capture of shadows with minimal diffraction as well as light emitted from the sample. On the other hand, lensless non-contact imaging involves placing the sample at a distance greater than 10 μm from the sensor, enabling the capture of diffraction patterns or emitted light from the sample. Lensless imaging methodologies can be further classified as either shadow-based or luminescence-based, depending on the source of the photons captured by the image sensor. Shadow-based imaging collects photons from an external light source, resulting in a shadow pattern cast by the sample. In non-contact imaging, this shadow pattern is a diffraction pattern with ripple-like bands at the edge of the shadow, as illustrated in Figure 1c, and is referred to as diffractive shadow imaging. Contact imaging, on the other hand, produces a geometric shadow with a clear edge and minimal diffraction, as shown in Figure 1f, and is termed geometric shadow imaging. Unlike shadow-based imaging, luminescence-based imaging collects photons emitted from the sample in the form of fluorescence, chemiluminescence (CL), electrochemiluminescence (ECL), and bioluminescence (BL). This imaging technique can be categorized as either contact-mode luminescence or non-contact-mode luminescence, based on whether the sample is in direct contact with the sensor surface.

### 2.2 Lensless non-contact imaging

Lensless non-contact imaging includes diffraction shadow imaging and non-contact mode luminescence imaging. Diffraction shadow imaging enables 3D imaging of the sample by capturing the diffraction patterns, providing qualitative results that can be further analyzed with autorecognition and counting algorithms. Lensless non-contact mode luminescence imaging, on the other hand, captures the luminescence emitted by the sample, providing higher optical contrast and quantified results in the form of light intensity, which can be used to calculate analyte concentration. Lensless non-contact mode luminescence imaging can be further divided into lensless fluorescence imaging and other lensless luminescence-based imaging techniques that use optic fibers to achieve non-contact-mode imaging with chemiluminescence, electrochemiluminescence, or bioluminescence.

Diffraction shadow imaging (Figure 1c), also known as digital inline-holographic microscopy (DIHM), uses a coherent or partially coherent light source to illuminate a sample to form scattered light, and the image sensor records the diffraction patterns generated from interference between the scattered light and the reference light [18]. These patterns, known as in-line holograms, can provide information on both the optical amplitude and phase of the scattered light from the sample, allowing for the reconstruction of a 3D image of the sample using reconstruction techniques [16].

Coherent light sources, such as lasers, are typically used for diffraction shadow imaging due to their ability to ensure high spatial resolution. Specifically, spatial coherence, which is determined by the aperture size of the illumination relative to the distance between the light source and sample, affects both the spatial coherence diameter at the sensor plane and the spatial smearing of the hologram [14]. Temporal coherence, which is determined by the temporal coherence length of the light source [16], also plays a significant role in determining the spatial resolution. For a scattered wave to be recorded at the sensor plane, the temporal coherence length should exceed the difference in optical pathlength between the scattered wave and the reference wave [14]. If the temporal coherence length is too short, it can limit the maximum angle of scattered waves contributing to the image, reducing the overall spatial resolution [14]. However, incoherent or partially coherent light sources, such as LEDs, are also being utilized for diffraction shadow imaging. This is because, in an on-chip configuration, partially coherent light sources are able to provide sufficient spatial coherence due to the close proximity between the sample and sensor and the relatively large distance between the light source and sample (i.e. *z*_1_ >> *z*_2_) which allows the light to behave as a point source [16]. Despite the shorter temporal coherence length of incoherent lights, the scattered light rays and reference light can still interfere at the sensor surface since the sample plane is close to the sensor plane [19].

To reconstruct 3D images from the obtained in-line holograms, there are different methods. While the conventional method utilizes iterative phrase retrieval algorithms that compute the Fourier transform of the holograms [16], deep learning-based reconstruction methods using trained neural networks have been developed in recent years, producing faster results with reduced artifacts [20, 21]. The training of the neural networks involves learning examples of holograms and the corresponding object fields. The trained neural networks can quickly reconstruct the phase and amplitude images of an object within a few seconds using only one intensity-only hologram [20], which is about 15 times faster than conventional iterative phase retrieval algorithms [21]. Another recently reported method involves using a passive optical processor consisting of transmissive diffractive layers to project the image of an object without any digital computation. Deep learning is used to design the diffractive layers in order to reconstruct in-line holograms and create an image at the speed of light [22]. Nevertheless, these reconstruction processes introduce additional computation or optical components; therefore, a simpler form of lensless imaging technique can be selected if 3D imaging is not required.

Diffraction shadow imaging has been applied in two main areas. The first area involves the detection and characterization of cells and microorganisms, such as red blood cells and various parasites, for diagnostic or environmental safety purposes [23–30]. An abundance of research has been conducted on diffraction shadow imaging, showcasing its numerous advantages over conventional lens-based systems, including higher throughput – the ability to capture thousands of micro-objects within a single digital image [31], rapid results that can be obtained within a minute [31, 32], automation capabilities, and accuracy that is comparable with or even surpasses conventional methods. Diffraction shadow imaging is also being explored for cell counting, viability analysis, and classification. For cell counting, the image sensor captures the shadows of cells, which are then automatically processed through a custom-developed algorithm to determine the cell count [33, 34]. By analyzing several parameters of the diffraction patterns, algorithms are employed to distinguish viable cells [34] as well as classify different cell types [35]. Most of these platforms utilize the optofluidic technique (Figure 1b), where a microfluidic device is used to manipulate the samples above the sensor arrays, to achieve easy and flexible sample handling [26, 29, 36]. The other area of diffraction shadow imaging application is cell motility analysis, where images of the sample are continuously taken at a specific frame rate of around 10 fps to track the cells on a 2D plane [15, 37] or in 3D via 3D reconstruction [38, 39]. Diffraction shadow imaging’s ability to record 3D motility patterns is a significant advantage, as cell motility patterns can appear similar in 2D but radically distinct in 3D [39].

Lensless on-chip fluorescence imaging (Figure 1d) uses fluorescence labels to obtain fluorescence images on the chip. The method involves the use of an incoherent light source as an excitation light to induce the emission of fluorescence. An absorption filter is placed above the image sensor to selectively block the excitation light while permitting the emitted light from the sample to pass through [40] to generate an image [41]. The requirement for an absorption filter between the sample and the sensor makes lensless fluorescence imaging a form of non-contact imaging method. Fluorescence imaging has proven useful for characterizing rare cells with a concentration of less than a few hundred per milliliter [42]. As previously discussed, conventional lens-based microscopes have limited FOV, whereas on-chip imaging provides a FOV that can span over several square centimeters, providing advantages for fluorescence imaging. Since fluorescence has low intensity and is non-directional, most lensless fluorescence imaging platforms require the sample to be in close proximity to the image sensor to maximize photon collection efficiency by capturing fluorescence signals emitted by the sample before they significantly diverge [43, 44]. To achieve close proximity, the thickness of the absorption filter should be minimized while maintaining sufficient background rejection. While conventional filters require a thickness larger than 10 μm to provide the desired amount of background rejection, silo-filters with metal lattice structures [45] have been developed to achieve comparable background rejection at thicknesses less than 10 μm.

Various lensless fluorescence imaging platforms have utilized a fiber optic plate, which is a bundle of optical fibers that transmits light from the sample to the detector, while passing through the absorption filter [46]. The use of a fiber optic plate allows the sample to be placed over 1 cm away from the image sensor, enabling the shift of the focal plane away from the image sensor surface, thereby protecting the absorption filter from overexposure to light and ensuring its longevity [46]. Additionally, the fiber optic plate provides thermal isolation between the sample and sensor to maintain optimal operating temperatures for the sample [41]. While the fiber optic plate may have similar densities on both sides with no magnification [47], a tapered geometry of the fiber optic plate where the top facet has a higher density of optical fibers than the bottom provides aberration-free magnification of approximately 2-fold, thereby enhancing the spatial resolution of the acquired image [41, 48]. The use of optic fiber bundles also enables non-contact imaging for other imaging techniques that utilize luminescence, including chemiluminescence (CL), electrochemiluminescence (ECL), and bioluminescence (BL), with the implementation being demonstrated in bioluminescence lensless imaging platforms as shown in (Figure 1e) [49–51].

### 2.3 Lensless contact imaging

Lensless contact imaging, characterized by the close proximity between the sample and sensor surface, can be achieved in geometric shadow-based modality and luminescence-based modality. Due to the infinitesimal *z*_2_, shadow-based imaging minimizes the diffraction patterns and eliminates the need for complex image reconstruction processes, making it a simpler form of lensless imaging than diffraction shadow imaging. Luminescence-based lensless contact imaging includes CL, ECL, and BL based methods, providing higher optical contrast compared to the shadow-based counterpart, with the ability to perform quantitative sensing of analytes in the sample.

In geometric shadow-based lensless contact imaging platforms (Figure 1f and g), the image is formed directly by the projection of light from the sample onto the pixels. In this case, spatial resolution is achieved by mapping different regions of the sample onto different pixels on the image sensor. Furthermore, this nature of contact imaging eliminates the requirement for coherence of the light source, distinguishing it from diffraction shadow imaging. In geometric shadow-based contact imaging, the distance between the sample and the sensor surface is minimized by directly placing the sample on the sensor array to reduce diffraction [19] and spatial overlapping between shadows of different objects [52], producing shadows that closely resemble the original objects [16]. By sensing the light transmitted through the sample, geometric shadow-based contact imaging captures the shadows that represent 2D images of the sample [19]. As biological objects are often partially transparent, the recorded shadows exhibit grayscale shades ranging from black to white, allowing different types of particles to be distinguished by matching their shadow patterns [16]. The platform developed by Imanbekova et al. in 2020, shown in Figure 1g, was the first to demonstrate three-dimensional (3D) measurement of the sample using oblique illumination in a geometric shadow-based contact imaging system [53]. Unlike diffraction shadow imaging, this platform does not require complicated image reconstruction processes to obtain 3D images.

In lensless contact imaging techniques that do not rely on shadows, luminescence-based methods utilize the sample-emitted light generated by CL, ECL, or BL to provide images with higher optical contrast capable of quantitative analyte sensing. The close proximity between the sample and sensor surface facilitates optimal photon collection during the imaging process. CL-based lensless contact imaging (Figure 1h) [54, 55] involves placing the sample on top of the image sensor surface and capturing the light emitted from chemical reactions that emit photons as excited molecules relax to their ground state [56]. These reactions typically employ labels such as luminol for chemiluminescent immunoassays or horseradish peroxidase or alkaline phosphatase for enzyme immunoassays. This imaging technique is versatile and can be used to quantify biomolecules ranging from toxins [54] to disease biomarkers [55], making it a powerful tool for food safety, diagnostics, and health monitoring. In BL-based lensless contact imaging (Figure 1i) [57], yeast or bacteria cells are genetically engineered to express recognition elements that interact with the analyte to activate the expression of luciferase enzymes, leading to the emission of bioluminescence. The bioluminescent cells are immobilized on or transferred to the active area of the image sensor, which enables the capture of light emissions from the cells. As the BL signals are directly proportional to the cell viability [50], an on-chip system that measures BL using lensless imaging techniques can serve as a powerful tool for detecting toxicity for drug research and environmental safety monitoring [58]. ECL-based lensless contact imaging (Figure 1j) [17] uses image sensors to capture light emission from an electrochemical process driven by an electric field that produces excited states through highly energetic electron transfer reactions in molecules at electrode surfaces [59]. Unlike CL reactions, which require catalysts to generate visible amount of light emission, ECL reactions use the electric potential difference to produce the excited state in a more controlled manner. Thus, ECL reactions can be considered more advantageous over CL reactions for use in lensless contact imaging.

In conclusion, lensless on-chip imaging simultaneously enables large FOV, high spatial resolution, and 3D imaging, while being portable and inexpensive. Particularly, the large FOV is ideal for high throughput sensing for tests that require analyzing large data sets, and the high spatial resolution enables the detection of small amounts of analytes. These advantages are highlighted for a wide range of biological sample analysis and lab-on-a-chip (LOC) applications. As outlined in Box 1, each lensless imaging method presents distinct advantages and limitations, and the optimal method selection depends on the intended application.

**Table:** 

**Box 1│ Classification of lensless imaging techniques**
Lensless imaging techniques are classified into contact imaging and non-contact imaging based on the distance between the sample and the surface of the image sensor. Compared to lens-based imaging, lensless techniques offer advantages such as decoupling of spatial resolution and FOV, wide FOV for high throughput sensing, 3D imaging capability, enhanced portability, and cost-effectiveness. ***Lensless contact imaging (z***_***2***_ ***≤ 10 μm)*** *.* This method captures geometric shadows or emitted light (CL, ECL, and BL) from the sample by placing it in direct contact with the sensor surface, resulting in high photon collection efficiency. Compared to geometric shadow imaging, the CL, ECL, and BL modalities provide the added benefit of analyte quantification but require additional sample preparation and handling. ***Lensless non-contact imaging (z***_***2***_ ***> 10 μm)***. This method captures diffraction patterns or light emission (CL, ECL, BL, and fluorescence) from the sample placed at a distance from the sensor. Diffraction shadow imaging is label-free and capable of reconstructing a 3D still-image as well as motility patterns of the sample by inferring phase information from the diffraction patterns, with the drawback being the requirement for adequate coherence of the light source and the complex image reconstruction processes. Lensless fluorescence imaging offers a larger FOV compared to conventional fluorescence imaging techniques but suffers from background noise caused by the excitation light. Non-contact luminescence imaging can utilize a fiber optic plate between the sample and image sensor to provide magnification and thermal isolation, with the disadvantage being the added cost and complexity. ***Advantages of lensless contact imaging over lensless non-contact imaging*** *.* Compared to lensless non-contact imaging, lensless contact imaging allows for a more compact configuration and offers higher sensitivity for analyte quantification due to its higher photon collection efficiency. Lensless contact imaging also offers a simpler alternative to non-contact imaging techniques that require complex image reconstruction processes.

## 3 Fundamentals and fabrication methodologies of microfluidics

The nature of fluid flow at micron-scale, while enabling the harnessing of new physico-chemical realms, is inherently associated with challenges in design and fabrication. The complex, and sometimes not fully understood physics of the (bio)fluids, combined with the heterogeneity of biological samples (e.g., viscosity, shear thinning, etc.) requires robust systems with tolerance for such variabilities. However, the physical limitations of fabrication at micron-scale pose the margin of error. Resolving this dichotomy requires a deep understanding of the limitations, advantages, and shortcomings of both foundational rules governing these systems, and the fabrication methods available to execute them. This section provides working principles and fundamental parameters of microfluidics that support their operation as well as various fabrication methods. Recent achievements in microfluidics have also been highlighted.

### 3.1 Fundamentals of microfluidic devices

At small scales, capillary forces are inevitable and can have a notable impact on rates of flow, even when other active pumping techniques are employed. However, these capillary events can also be united with other effects, such as electrochemical and electrostatic forces, to create a toolkit to regulate the flow in microchannels independent of external sources. This can be further complemented with flow control components, like hydrophobic capillary stop valves, which are widely used in centrifugal and pressure-driven microfluidics, to determine which sections of the microfluidic chip fill first, based on their distinct burst pressures [60–63].

In general, flow in microfluidic devices is regulated by various components such as capillary pumps, flow resistors, and different types of valves. Proper design of flow compartments, including inlets and outlets is thus critical for efficient flow of liquids in microchannels. A patterned reaction chamber is also essential for capturing desired analytes during biological assays. To avoid corner flow, inlets can be designed with no features and by directly applying liquids to a narrow tube that protrudes laterally outside the edge of the surface of the chip. Reservoirs can range in size from very small (in the picoliter range) to very large (up to 100 μL) depending on the intended application. Reaction chambers are the sections in a capillary-driven flow microfluidics, capillaric microfluidics (CM), in which the main biochemical assays occur, and their design should consider the desired analyte concentration, liquid transport within the CM, and user-convenience of the CM [60, 64].

The correlation between capillary pressure, contact angle, and microchannel size, is described by the Young–Laplace equation, as follows [65]:

$$P=-\gamma \left[\frac{\cos\theta_{t}+\cos\theta_{b}}{h}+\frac{\cos\theta_{l}+\cos\theta_{r}}{w}\right]$$

This equation takes into account the surface tension of the liquid in the microfluidic channel (*γ*), the height (*h*) and width (*w*) of the channel, and the contact angles (*θ*_*r*_, *θ*_*l*_, *θ*_*b*_, *θ*_*t*_) at the right, left, bottom, and top walls, respectively. Using electrical comparations, the flow resistance (*R*) inside a microfluidic channel can be explained as [60]:

$$R=\frac{\Delta P}{Q}=\frac{12\eta L}{h^{3}w}{\left[1-0.630\frac{h}{w}\right]}^{-1}$$

where Δ*P* represents the variation in pressure within the small channel with the flow rate *Q* of liquid, while *L* indicates the extent of the liquid present in that channel. The passage describes how the flow rate of a liquid in a rectangular microchannel is affected by the variation in capillary pressure throughout the microfluidic channel and the length of the liquid in it. Altering the size of the microchannel can impact both the pressure inside the channel and the resistance to the flow of fluid. For instance, if the height of a rectangular microchannel is much smaller than its width, the capillary pressure is proportional to 1/*h*, while the flow resistance is proportional to 1/*h* ^3^. This means that decreasing the microchannel height increases capillary pressure, but it also significantly increases flow resistance, resulting in an overall reduction in flow rate of 1/*h* ^2^ and flow speed of 1/*h*. Capillary pumps play an essential role in CMs by drawing adequate sample and reagents to complete an assay and concurrently serving as excess reservoirs. To maintain a consistent flow rate in CMs, capillary pumps need to possess small characteristics, which can be as narrow as just a few micrometers, to produce sufficient capillary pressure that propels the flow forward [60, 66].

However, there are numerous obstacles that must be addressed to make CMs a feasible option for convenient diagnostics in point-of-need settings. Currently, most CMs with fluorescence recognition use conventional microscopes, but there are no established solutions for long-term reagent storage (e.g., pouches) or metering valves to reduce the dependence on pipetting. To make CMs more user-friendly and practical, they need to be integrated with optics and handheld readers and combined with simple colorimetric and luminescence readouts [67, 68].

### 3.2 Fabrication methodologies

**Figure 2:**
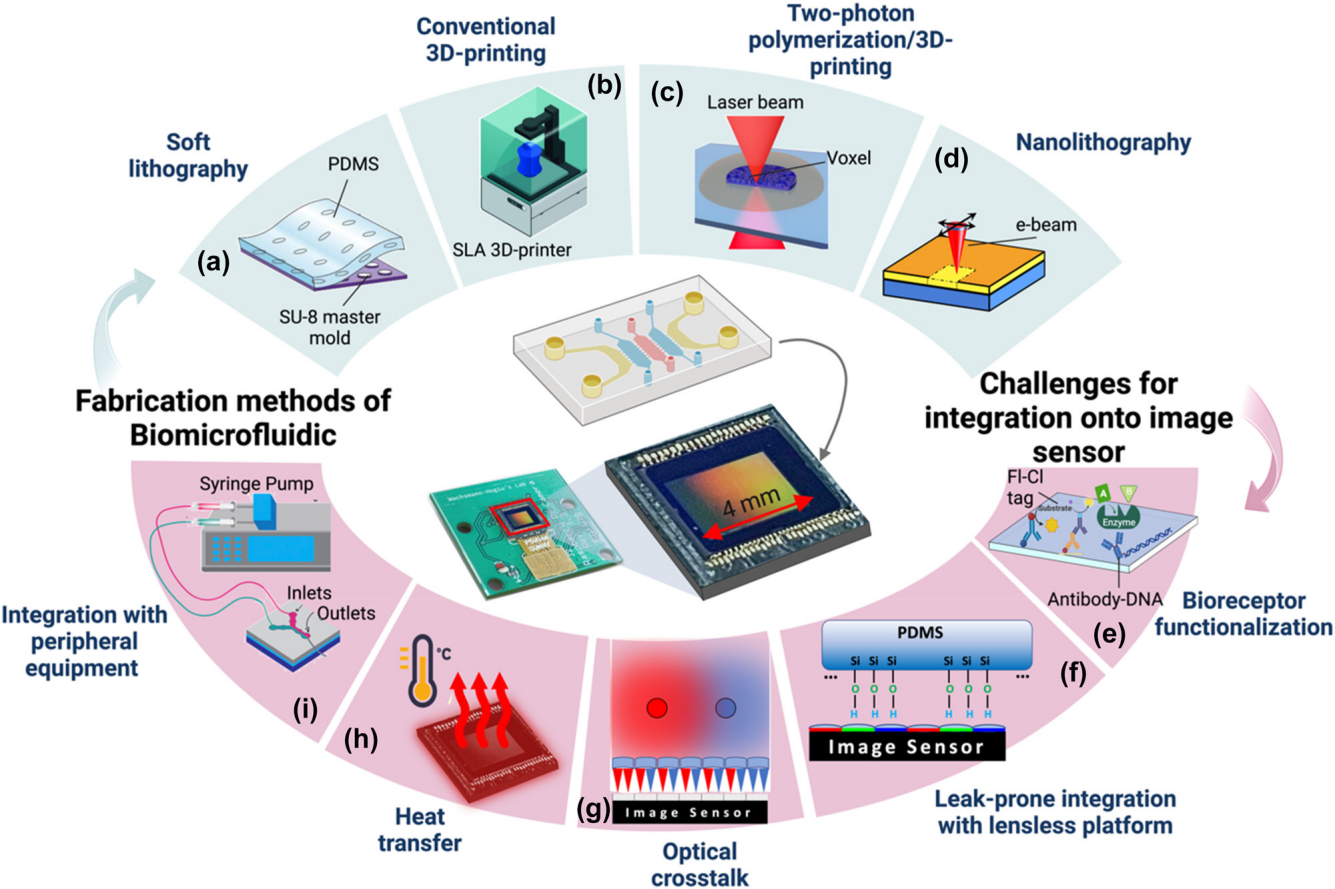
Fabrication of biomicrofluidics and integration into a lensless platform: *(Top) Fabrication methods of miniaturized biomicrofluidics*. (a) Soft lithography. Allows replicating the patterns of a hard substrate (e.g. silicon wafer as a mold) in a soft polymer, like PDMS *(modified with permission from* [69]). (b) Conventional 3D-printing. Modern desktop 3D printers can print channels well below 100 µm (down to 30 µm) in dimensions. (c) Two-photon polymerization 3D-printing. Utilizing non-linear absorption of two photons to create a smaller focal space (voxel) in a photo-polymer that can be as small as 0.2 µm. (d) Nanolithography. Employs techniques such as electron-beam and focused ion-beam, allowing it to achieve spatial resolutions as small as 10 nm and provide tight control over handling of picolitre volume of liquids. *(Bottom) Challenges for integration of biomicrofluidics and image sensor.* (e) Bioreceptor functionalization. The surface of the sensor needs to be first functionalized for the detection of a specific analyte of interest. (f) Leak-prone integration with lensless platforms. When integrating with lensless platforms, bonding the microfluidic chip to the imaging sensor’s surface can be challenging, as the semi-sphere microlenses on the surface create an uneven surface (g) Optical crosstalk occurs when light from the same point of the sample is detected simultaneously by different pixels. This leads to artifacts and lower image quality. (h) Heat transfer; the proximity of the sensor to the microfluidic chip significantly increases the heat transfer from the sensor to the chip (i) integration with peripheral equipment. Integration into peripheral equipment to manipulate the liquid sample.

The miniaturization of microfluidic devices has gained tremendous attention in recent years, as it offers numerous benefits such as reduced reagent consumption, improved sensitivity, and increased throughput. However, the integration of miniaturized microfluidics with lensless platforms remains a significant challenge. This section aims to provide an overview of various fabrication methods used to develop miniaturized microfluidic devices and discusses the challenges associated with integrating them with lensless platforms, as shown in Figure 2.

One of the main requirements of microfluidic devices is the ability to handle liquids at length-scales below 100 µm, in at least one dimension. This would necessitate a path, often an enclosed channel, for the liquid to flow in. Given the practical limitations of fabrications at this micro-scale, a lot of initial microchips were made using techniques borrowed from the semiconductor industry, hence the use glass and silicon wafers for first generation of microfluidics. With introduction of soft-lithography, the whole field shifted towards using silicones, and pre-dominantly poly-dimethyl siloxane, or PDMS, to fabricate these systems. The unique opto-mechanical properties of PDMS, combined with its biocompatibility, have turned it into the dominant material of choice for fabrication of microchannels, at least in academic works [70, 71]. Soft lithography allows one to replicate the patterns of a hard substrate (e.g. silicon wafer) in a soft polymer, like PDMS (Figure 2a). This means, creation of a microchannel is primarily dependent on creating a negative mold with dimensions in the micron range. For years, photolithography was the gold standard for creating molds for microfabrication. However, the need for a clean-room, expensive equipment and reagents (e.g. photo-resists), and expert personnel led to strong motivations to look for substitutive solutions to create molds [72, 73]. Not surprisingly, for any geometry that can be resembled by existing materials, like helical paths resembling twisting wires, the easiest and cheapest option is to use the existing material as the mold [74].

Despite their merits, such techniques are limited to creating 2D geometries, with limited heights, and often a uniform height for the whole mold. These limitations fueled the search for techniques to adapt 3D printing technologies with microfluidics fabrication (Figure 2b). However, for years, 3D printers have been unable to print structures with high enough spatial resolution (small enough details) to be amenable to microfluidics applications [75]. With recent advances, modern desktop 3D printers can print channels well below 100 µm (down to 30 µm for SLA 3D printers) in dimensions. This makes them a great candidate to both print a fully enclosed microfluidics system, and to make molds to be later replicated in PDMS. This allows for creation of more complicated chain of microchannels, capable of performing multiple complex tasks on a single chip [76].

One major drawback of using parts made of these polymers for PDMS casting though is their chemical interference. Most commercially available photo-resins contain components that hinder the curing process of PDMS, causing difficulties in replicating structures in the elastomer material with consistency. The platinum-based catalyst in PDMS (Sylgard 184), facilitates crosslinking of vinyl-terminated oligomers via hydrosilylation. However, certain compounds like tri-organophosphite, maleate, fumarate, and β-alkynol, can impede the platinum-catalyst and prevent PDMS curing by inhibiting the catalyst either through their strong affinity for it or by sequestering it in small droplets, either reversibly or irreversibly. In addition, 3D-printed parts release a range of substances into the solution, such as polyethylene glycols, diethyl-phthalates, unreacted monomers, and phosphine-oxide photoinitiators, which can also hinder the catalyst, and hence prevent PDMS from fully curing on the mold. This can often be rectified by post-treatment of 3D-printed parts through exposure to UV radiation, thermal treatment, rinse with solvents, silanization, or adding a coating layer [77].

The growing ambition to integrate more geometries on every microfluidics chip, further pushed the field of 3D printing to incorporate technologies beyond traditional methods, like stereolithography. One such technology is utilizing non-linear absorption of two photons to create a smaller focal space in a photo-polymer (Figure 2c). This photo-polymer has minimal absorption at the wavelength of the pulsed laser, thus no polymerization happens in the focalization cone. However, at the focal point, the photo-polymer can receive two simultaneous photons in a small volume, named “voxel”. This triggers a free-radical chemical reaction, converting the liquid monomer to a solid polymer within the voxel. By using the appropriate optical elements and polymer formulation (e.g. photo-initiators, photo-absorbers, etc.), the voxel size can be as small as 0.2 µm. This makes two-photon 3D printing an ideal technology for high-resolution fabrication [78].

The use of nano-lithography (Figure 2d), a process to create nanometer-scale patterns on a surface, opened the doors to a new chapter in LOC fabrication and fluid handling. Unlike traditional UV-based bottom-up techniques in photo-lithography, nano-lithography employs techniques such as electron-beam and focused ion-beam, allowing it to achieve spatial resolutions as small as 10 nm. Such small geometries and their resulting intricate structures provide tight control over the handling of picoliter volume of liquids [79, 80].

### 3.3 Recent advances in microfluidics

Biomicrofluidics is a rapidly evolving interdisciplinary field that couples biology and fluid physics at the scale of microconfinement. In recent years, biomicrofluidics research has been employed in various areas, including lab-on-a-chip, diagnostics, tissue engineering, and drug delivery.

Perhaps the most noteworthy application of biomicrofluidics relates to the lab-on-a-chip device, a device that integrates various laboratory functions on a single chip of only millimeters to a few square centimeters to enable automation and high-throughput analysis by using just a few micro-droplets of physiological fluids. The field has witnessed numerous breakthroughs in the development of miniaturized biosensors for rapid and accurate pathogen detection for diseases such as HIV, malaria, and for cancer diagnostics. They have also made significant contributions to various laboratory procedures, including DNA sequencing, hormone detection, and protein analysis [8, 81–87].

Microfluidic methods can also be advantageous in the blood cell separation process, since they can be utilized to effectively separate various components of blood, including red blood cells, white blood cells, platelets, and lipid particles. Moreover, microfluidic systems, due to their small size and transportability, are well-suited for point-of-care or decentralized testing of biomarkers. Applications such as real-time sampling and measurement of tissue biochemistry, C-reactive protein detection for monitoring inflammation in the body, analysis of biomarkers relevant to kidney disorders, and detection of glucose-cholesterol- uric acid by immobilizing different assay reagents, have been explored [86, 88–92].

In the realm of tissue engineering, biomicrofluidics has been used to create dynamic microenvironments that closely mimic the physiological conditions found in living tissues. Additionally, biomicrofluidics has the capability to simulate the 3D structure, mechanical properties, and biochemical microenvironment that cells experience in a living organ and cannot be simulated using conventional 2D cell cultures. These mentioned microenvironments can be utilized to control cell behavior, such as cell proliferation, differentiation, and migration. Microfluidic systems have also enabled the creation of perfusable microvasculature networks that can deliver nutrients and oxygen to cells, further advancing the development of artificial tissues and organs (Organ-on-a-chip) such as lung, cardiac muscle tissue, brain, liver, kidney, gut, and skin [93–102].

Moreover, biomicrofluidics have shown tremendous potential in the development of innovative drug delivery systems mainly due to the ability to control properties such as size, shape and structure, surface engineering, and elasticity of drug delivery systems. Using microfluidic device, nanoparticles and microparticles can be synthesized to precisely deliver drugs to targeted areas in the body. Thus, different applications of microfluidic techniques in drug delivery, including double and multiple emulations (forming and stabilizing droplets within another droplet), protein-based (such as gelatin and collagen) nanocarriers, lipid-based (such as liposomes and niosomes) nanoparticles, and polymeric and hybrid nanoparticles, have been investigated [103–114].

As biomicrofluidics continues to advance, it holds immense potential to revolutionize medicine and improve patient outcomes. Further research in this area is crucial to unlock new possibilities in the development of diagnostic tools, drug delivery systems, and tissue engineering applications. To make them practical for real-world applications, advancements in platform fabrication, analytical methods, and detection techniques are necessary. Collaborations of researchers from diverse backgrounds and areas of expertise with the purpose of biomicrofluidics development have seen increasing success in recent years, and thus microfluidics gains more acceptance in the life sciences beyond engineering and method development, this trend is expected to continue to grow in the near future. Improved and consistent techniques for creating multiple emulsions will increase the complexity of particles that can be synthesized, leading to more precise delivery and administration of drugs. This will improve the ability to encapsulate molecules and ultimately result in more tailored therapeutic interventions. Lastly, by combining optics and microfluidics, it is possible to introduce novel capabilities without sacrificing integrability or compactness.

### 3.4 Path from micro- to nano-fluidics

Nanofluidics is a field that involves investigating and utilizing fluids within and near structures that have dimensions on the nanoscale (less than 100 nm) [115]. While not a completely novel field, aspects related to fluid behavior at this scale have been intermittently addressed by scientists in areas like membrane science, colloid science, and chemical engineering for a number of years [116, 117]. However, nanofluidics is currently receiving more significant focus due to advancements in nanofabrication, which are driving the recent expansion of this field [116, 117].

As the channel dimensions and fluid volume transition from micro to nano scales, there is a reduction in the size of the nanofluidic device. Consequently, there is a necessity to correspondingly enhance the spatial resolution of the image sensor in a biosensing system that utilizes the image sensor as an optical transducer. As the spatial resolution of image sensors is currently restricted by the size of the pixels on the sensors, the pixels have been steadily decreasing in size over the past two decades. The miniaturization of pixel size is restricted by the performance degradation that accompanies the pixel shrinkage. As the pixels shrink, they collect less light, making photon collection efficiency crucial. However, as pixel size approaches the wavelength of visible light, significant diffraction effects occur at the pixel and the ray-tracing model cannot sufficiently describe the light wave propagation [118]. This results in increased spatial optical crosstalk, where adjacent photodiodes receive more photons due to diffraction, leading to incorrect signals at neighboring pixels, reduced spatial resolution, and reduced color accuracy [118, 119]. Additionally, the light collected by an adjacent pixel is lost from the intended pixel, resulting in decreased photon collection efficiency of the intended pixel [119]. Reducing pixel size also means smaller photodiode and reduced transistor dimensions, which can lead to increased dark noise, slower readout speed degradation, and reduced full-well capacity- the maximum amount of charge that can be stored in a pixel [120].

To overcome the degrading pixel performance described before, several technologies have been developed, including customizing the pixel architecture, microlens optimization, and light guiding structures. BSI, deep trench isolation, vertical transfer gate, multi-thickness gate oxide, and switchable conversion gain are addressing issues related to the pixel configuration and architecture to improve full well capacity, signal-to-noise ratio, readout speed, and dynamic range [118, 120, 121]. As the microlens array enhances photon collection efficiency by concentrating the incident light, further enhancements in photon collection efficiency can be achieved by optimizing the radius of curvature of the microlens to focus the maximum amount of light onto the photodiode [118]. Light guiding structures from the microlens to the photodiode can lead to higher photon collection efficiency by confining and directing more light towards the photodiode via total internal reflection [119]. As technological advancements continue to mitigate the pixel performance degradation associated with pixel shrinkage, the pixel size miniaturization has reached 0.7 μm.

Given the increase in surface area to volume ratio as objects shrink, nanochannels exhibit a significant surface-charge-induced transport effect. This phenomenon, guided by electrostatics theory in liquids and electrokinetic effects in nanochannels, lays the foundation for ion separation. The selectivity of this charge-based transport is most pronounced when the Debye screening length matches the smallest dimension of the nanochannel’s cross-section. This results in an aperture at the nanometer scale that primarily contains counterions. These distinctive characteristics play a role in the charge-dependent distribution of biomolecules at the boundary between microchannels and nanochannels. Additionally, these properties allow for the separation of biomolecules based on their charge at the interface between microchannels and nanochannels. The associated energy barrier also facilitates size-based separation when biomolecules and nanoconstrictions share similar dimensions [122–124]. Another important contribution of nanofluidics is to the fundamental characterization of liquids and small molecules, such as in biophysics and fluid mechanics. In this context, small molecules enclosed within extremely small volumes in nanofluidic systems are exposed to controlled forces for high-resolution measurements [122, 123]. Such capabilities can be amplified when combined with structures like nanopores and nanowires. Based on these intriguing physical principles, these structures can detect biomolecules with high sensitivity, without the need for labels, and in real-time, suggesting their significant potential for life sciences applications [122, 123, 125].

The Navier–Stokes equations explain the fluid flow. However, the boundary for the equation’s applicability is determined by the fluid’s molecular dimension, which is typically around 1 nm [126]. This scale serves as the lower limit for defining fluid viscosity (*η*). In macroscopic fluid mechanics, the kinematic viscosity (*ν* = *η*/*ρ*), with *ρ* representing mass density, acts as a diffusion coefficient for the fluid’s momentum. Based on the microscopic origin of diffusion, it’s essential that the time necessary for momentum to disperse across the system exceeds the timeframe of molecular motion. Recent advancements in fabrication technology have empowered researchers to overcome the challenges of developing nanofluidic systems, allowing the development of artificial devices with structures as small as a single water molecule (approximately 3 Å). At this scale, factors such as water structuring due to surfaces, memory effects, and various subcontinuum phenomena become influential. Interestingly, when dealing with water flow below 10 nm in length, and even with fluid velocities reaching 10 m/s, the Reynolds number stays below 0.1 [127]. Consequently, in nanofluidic systems, inertial effects can be safely disregarded, and the fluid flow is accurately characterized by the simplified Stokes equation:

ηΔv+f=∇p

Here, *p* indicates pressure and *f* represents a body force, which might result from factors like the application of an electric field [127].

In the realm of nanoscale fluid flows, certain microscopic factors that are often negligible in the Navier–Stokes equation become very important. Notably, molecular binding energies of fluid-fluid and fluid-surface interactions play crucial roles [128, 129]. These microscopic factors bear a direct correlation with macroscopic variables comprising temperature and the external driving force within the flow system. System temperature, which indicates thermal agitation among fluid molecules, can weaken fluid-surface bindings under elevated temperatures [122, 130, 131]. Likewise, a substantial driving force can enable fluid molecules to overcome surface attractions [132, 133]. Conversely, at lower temperatures and with diminished driving forces, the contest between the energies associated with fluid-fluid and fluid-surface interactions gains significance [132, 134]. The interplay of these parameters across different scales determines distinct flow regimes, each characterized by unique mechanisms of how these parameters influence fluid motion [127].

It is widely recognized that the resolving capability of conventional photolithography is restricted by the wavelength of the incident light, which significantly surpasses the critical dimensions needed for nanofluidic investigations. Consequently, it is a logical approach to utilize alternative lithography methodologies with nanometer-level precision for producing nanofluidic devices. Up until now, several nanolithography methods have emerged with the ability to surpass the light diffraction limitations seen in typical photolithography [122, 123]. These techniques comprise electron beam lithography (EBL) [135–137], focused ion beam (FIB) [138, 139], nanoimprint lithography (NIL) [140, 141], interferometric lithography (IL) [80, 142, 143], and sphere lithography (SL) [144, 145]. The first two, EBL and FIB, are effective techniques for generating single or small-scale nanochannels through direct writing procedures. Meanwhile, the remaining three methods, NIL, IL, and SL, are typically employed for constructing arrays of larger-scale nanopores or nanochannels [146, 147].

While nanolithography methods offer the capability to produce diverse nanostructures, the prevailing techniques for nanofabrication still heavily lean on standard MEMS (micro-electro-mechanical systems) approaches [147]. This preference is driven by their efficiency in large-scale production and cost-effectiveness, capitalizing on their capability for wafer-scale processing. The fabrication processes rooted in MEMS typically involve defining structures using conventional photolithography and shaping these structures through a sequence of additive (deposition) and subtractive (etching) steps. Even though standard photolithography tools in research settings are not directly equipped to create features at the nanoscale, precise manipulation within the precisely defined deposition and etching procedures can yield structures with nanoscale depth and/or width. There are five distinct fabrication methods that are based on MEMS. Sacrificial layer release and etching, along with bonding, are commonly employed for producing 2D planar nanochannels with a low aspect ratio. On the other hand, the remaining three techniques, which involve etching and deposition, edge lithography, and the spacer technique, are better suited for crafting 2D vertical nanochannels with a higher aspect ratio [122, 123, 148, 149]. Recently, the utilization of nanomaterials in the creation of nanofluidic devices has gained substantial traction as a favored fabrication approach. Diverse nanomaterials, spanning from ion-selective polymers with molecular-scale pores to nanoporous membranes, and ranging from zero-dimensional nanoparticles to one-dimensional nanowires and nanotubes, have been harnessed to construct nanofluidic devices, capitalizing on their inherent nanometer-scale characteristics [147].

The realization of nanostructures extends beyond mere fabrication technologies, demanding integration into the macroscopic realm. Within a microfluidic chip, nanochannels can be meticulously designed to gradually transition from nanometer to micrometer and even millimeter scales. Furthermore, special attention is placed on the choice of materials for constructing nanochannels, seeking specific attributes such as hydrophilicity to facilitate effortless capillary-driven channel filling, non-conductivity for precise high-resistance measurements, structural integrity to minimize surface deformations and endure high pressures, transparency to accommodate luminescence experiments, and the potential for subsequent biochemical surface modifications [122, 123, 150].

The majority of applications in the field of nanofluidics will predominantly involve chemical or physical analysis, necessitating some form of detection method [151]. Particularly in chemical analysis, detecting low concentrations within small detection volumes poses a significant challenge. For instance, a 100 × 100 × 100 nm cube (10^−18^ L) would only hold a mere six molecules at an analyte concentration of 10 μM, demanding the use of costly single-molecule detection techniques [122]. Addressing this issue can be approached through the implementation of spatially parallel structures on a large scale, possible through micromachining processes, while maintaining a high level of spatial uniformity. Alternatively, a more robust solution is provided by continuous flow structures like DNA separation devices. In this particular scenario, the nanoscale structuring aids in separation, while the time integration assists in detection [122, 123, 152].

In the realm of (bio)physical analysis, spatial information often proves essential, such as discerning the positioning of regulatory factors on DNA or even more ambitious goals like identifying the location of individual base pairs. Near-field optical microscopy emerges as a potential avenue for achieving the former objective, currently offering a resolution of around 50 nm. This method aligns well with integration into nanofluidic systems. Notably, conductometric detection is also approaching the realization of the former goal, enabling the identification of single bases during the translocation of DNA through a nanopore [122, 123]. More recently, a method called convex lens-induced confinement (CLiC) microscopy has been utilized to achieve detection at the level of individual particles. This technique has been employed to isolate and measure the diffusive paths and fluorescence signals of separate nanoparticles, which are trapped within microwells for extended periods [153]. It was utilized to study the dimensions and loading capacity of lipid nanoparticle (LNP) carriers, carrying silencing RNA (siRNA), depending on lipid composition, solution acidity, and drug encapsulation.

Within nanochannels, molecules maintain a close proximity to the channel walls, making the adsorption of proteins, especially those of an amphiphilic nature, more likely to happen. To counteract this phenomenon, it’s commonly suggested to employ a layer of polyethyleneglycol (also known as polyethyleneoxide) that can be applied through vapor deposition. In situations involving two-phase flow in the presence of proteins, a challenge arises due to potential denaturation at the interface between the liquid and water. Additionally, maintaining stability for proteins and DNA necessitates the avoidance of high shear rates [122, 123, 154]. Furthermore, an intriguing occurrence witnessed in nanochannels during two-phase flow involves the creation of negative pressure caused by capillarity. This pressure was detected within devices where the slender roofs of the channels were distorted due to the negative pressure generated by a capillary liquid plug [124, 155].

## 4 Requirements and challenges for integration of microfluidics with lensless optical image sensors for biosensing

Previous sections highlighted recent advancements in biomicrofluidics and lensless image sensors. In the following section, we will explore opportunities for integrating microfluidics and image sensing into a single platform, where a microfluidic device is placed directly on top of an image sensor. The synergistic integration of optics and fluidics leads to the emergence of optofluidic systems, acquiring enhanced sensing performance and oftentimes a more compact design [156, 157]. A lensless contact imaging system integrated with a microfluidic component is a form of optofluidics, combining the advantages of both fields, creating a portable, inexpensive, and simple platform capable of handling small sample volumes to produce high-throughput results with good spatial resolution. The miniature sizes of image sensors and microfluidic devices are compatible with each other, and the microfluidic device’s ability to manipulate small sample volumes in multiple channels enables multiplexed on-chip imaging, which can be challenging without microfluidics. In the following section, we will be discussing the challenges, advancements, applications, and requirements related to bioreceptor functionalization, integration of microfluidic chips onto the lensless system, fluid flow, experimental conditions, and data transfer.

### 4.1 Bioreceptor functionalization

Microfluidic integrated shadow-based lensless contact imaging enables cytometry [158–161], where images of cells of interest flowing through the microfluidic channel are captured by the image sensor. The recorded images can be used for cell counting or characterization. On the other hand, luminescence-based lensless contact imaging can be used for biosensing applications by enabling quantitative measurements of analytes of interest based on the correlation between the luminescence signals and the concentration of the target analyte. To achieve microfluidic integrated luminescence-based lensless contact imaging, analyte-specific light-emitting reactions must take place in the microfluidic channel. This requires careful selection of a suitable reaction and functionalization of the chip with appropriate biorecognition elements.

#### 4.1.1 Methods for functionalization

In the process of CL reactions, the CL substrate is oxidized, producing an intermediate that decomposes to generate an excited luminogenic species that emits light when it decays to the ground state. Luminol is the most widely used CL substrate, which can be directly oxidized by various oxidizing agents such as hydrogen peroxide (H_2_O_2_) in an alkaline solution to produce blue light with maximum intensity centered at around 425 nm [162]. Metal ions or metalloproteins such as hemoglobin and peroxidase (e.g. horseradish peroxidase) are used as catalysts to accelerate the reaction and enhance the light intensity [162]. Another commonly used CL substrate is the ruthenium complex, which operates by oxidizing [Ru(bpy)_3_]^2+^ to [Ru(bpy)_3_]^3+^, followed by reducing [Ru(bpy)_3_]^3+^ to an excited state that decays to the ground state while emitting orange light centered at around 610 nm [163].

CL biosensing methods on a microfluidic integrated luminescence-based lensless contact imaging platform include CL immunoassays, DNA hybridization assays, and enzymatic sensors. CL immunoassays can be performed in different formats [164]. First, direct assays require immobilizing the sample on the sensor surface and using CL-labeled antibodies to directly bind to the antigens of interest. Second, indirect assays first immobilize the sample on the sensor surface, followed by using unlabeled primary antibodies to bind to the antigens of interest and then using CL-labeled secondary antibodies to bind to the primary antibody. Third, sandwich assays involve immobilizing capture antibodies on the sensor surface, followed by adding the sample where target antigens bind to the capture antibodies, and then adding CL-labeled detection antibodies to bind to a different epitope on the antigen. Fourth, competitive assays use a known amount of labeled antigens along with unlabeled target antigens to competitively bind to a limited amount of antibodies, producing a CL signal that is inversely proportional to the number of target antigens present in the sample. In the first three formats, blocking buffer with non-reactive proteins such as bovine serum albumin and casein is added after the immobilization step to saturate all unbound sites. Another type of CL immunoassay is the magnetic-bead-based CL immunoassay, which uses magnetic beads coated with antibodies as solid support to capture the target analyte in a sample. The magnetic beads enable easy removal of the unbound CL probes using an external magnetic field [165]. DNA hybridization CL assays rely on Watson–Crick base-pairing [166], and can be employed in sandwich and structure-switching formats. In the sandwich format, capture probes are immobilized on the sensor surface and the target ss-DNA is hybridized with capture probes and then hybridized with CL probes. Alternatively, the structure switching format entails the immobilization of CL-labeled hairpins on the sensor surface followed by hybridization of the target ss-DNA with the hairpins, leading to a modification of the CL signal. For CL enzymatic measurement, a reagent containing a CL substrate is needed to react with the enzyme to produce CL signal, along with other components to enhance the signal. To initiate the reaction, the sample and the reagent should be mixed thoroughly. The resulting light emission is proportional to the enzymatic activity in the sample.

ECL in biosensing applications is most prominently generated by a co-reactant process. This process involves an electric potential gradient produced by the electrode’s resistance in an electrolyte solution containing luminophore and co-reactant. Both the luminophore and co-reactant species are oxidized or reduced, followed by the decomposition of these intermediates into highly reducing or oxidizing species. Electron transfer between these species and the oxidized or reduced luminophore generates the excited state of the luminophore, which decays radiatively to the ground state [167]. The most commonly used luminophores are luminol, ruthenium complex, and nanomaterials such as quantum dots (QDs), with the corresponding co-reactants being H_2_O_2_, Tri-n-propylamine (TPA or TrPA), and peroxydisulfate (S_2_O_8_^2−^), respectively [168]. The luminol-H_2_O_2_ ECL system has the advantage of a relatively low working potential and coupling with enzyme labels allows for the detection of the enzymatic substrate. However, luminol’s reaction is irreversible, and the ECL intensity varies according to the system’s pH. On the other hand, the Ru(bpy)_3_^2+^-TPA ECL system has reversible luminophores and high ECL efficiency, with a relatively low working potential [168]. Due to its tunable luminescence properties based on the size of the QDs, the QDs-S_2_O_8_^2−^ ECL system is receiving increasing attention in biosensing research.

In ECL biosensing methods, recognition elements such as antibodies, single-stranded DNA (ss-DNA), and enzymes are used to selectively bind to the target analyte, and the resulting biochemical interactions are translated into quantifiable ECL signals that can be used to calculate the concentration of the target analyte. Similar to the CL counterpart, ECL biosensing methods include ECL immunoassays, DNA hybridization assays, and enzymatic measurement. In ECL immunoassays, immunoreactions are typically conducted on the electrode surface and categorized into the same formats as in CL immunoassays. As the most common format, the sandwich ECL immunoassay is achieved by first immobilizing the primary capture antibodies on the surface of the working electrode, followed by binding the target antigens to the capture probe and then binding the ECL-labeled secondary antibodies to the target antigens [168]. ECL measurement is conducted after washing out the unbound ECL probes. Similar to CL immunoassays, magnetic beads can also be used in ECL immunoassays where the capture antibodies are immobilized on the magnetic bead surface [169]. DNA hybridization ECL assays employ similar formats as the CL counterpart, except the immobilized DNA strands are located on the electrode surface. ECL enzymatic measurement involves the integration of an enzyme catalytic reaction with ECL detection, whereby the co-reactants involved are either a coproduct or cofactor of the enzymatic reaction [168]. The luminol-H_2_O_2_ system represents one of the most common ECL enzymatic biosensing methods, primarily employed in the determination of the concentration of enzymatic substrates such as glucose and uric acid [17]. In this system, oxidase catalysts facilitate the reaction between enzymatic substrates and dissolved oxygen, resulting in the production of H_2_O_2_. The H_2_O_2_ subsequently reacts with the electrochemical oxidation of luminol, leading to photon emission.

To select a biosensing method for functionalizing the microfluidic chip in an integrated luminescence-based lensless contact imaging platform, it is important to evaluate the advantages, suitability, and drawbacks of each option. CL methods benefit from the simplicity of not requiring an electric field and therefore avoiding the need for additional integration of electrical components into the microfluidic chip. However, for a luminescence-based lensless contact imaging platform, it is crucial to consider the spatial and temporal control of the sample in the microfluidic channel for capturing light emission at the image sensor’s active area. ECL methods provide superior temporal and spatial control of light emission by allowing applied potentials to be switched on and off, controlling the reaction timing and confining it near the electrode surface.

#### 4.1.2 Challenges for functionalization

Functionalization in microfluidics (Figure 2e) is the process of modifying the surface properties of microchannels and microdevices to improve their performance and functionality. The functionalization of microfluidic systems is a critical step in the development of microfluidic devices for a variety of applications, including chemical and biological analysis, drug discovery, and biomedical diagnostics. Despite its importance, functionalization in microfluidics presents several challenges that must be addressed to ensure the reliability and reproducibility of microfluidic systems. One of the main challenges is the need for control over surface chemistry and morphology to achieve the desired surface properties. This requires the use of sophisticated techniques, such as plasma treatment, chemical vapor deposition, and self-assembled monolayer deposition. Another challenge is the stability and durability of the functionalized surfaces, which can be affected by factors such as temperature, pH, and mechanical stress. Additionally, the integration of multiple functionalized surfaces in a single microfluidic device can pose technical challenges, such as the prevention of cross-contamination and the optimization of fluidic transport. Addressing these challenges requires a multidisciplinary approach that combines expertise in materials science, chemistry, and microfluidics [170].

### 4.2 Integration of microfluidics with lensless platforms

**Figure 3:**
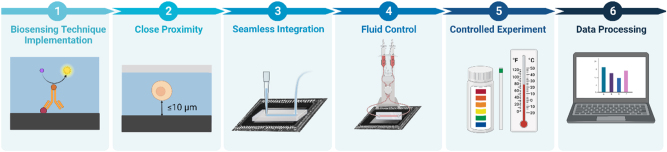
Key requirements to achieve biomicrofluidic integrated lensless contact imaging. (1) Biosensing technique implementation and the functionalization of the sensor surface. (2) Close proximity between the sample and the sensor surface. (3) Seamless integration between the microfluidic device and the sensor. (4) Fluid control to deliver the sample and confine the reaction to the active area of the sensor surface. (5) Physical experimental condition maintenance of the pH and temperature. (6) Data processing to analyze the data and potentially increase the spatial resolution of the acquired image.

As discussed earlier, microfluidic lensless contact imaging has proven to be a versatile technique for both dynamic and static imaging of liquid samples, as well as for performing chemical and biological sensing. However, to further become a mainstream technology with commercial applications, further research and development is needed to extend its capabilities. In the following discussion, we will examine key constraints, requirements, and challenges for the design of microfluidic integrated lensless contact imaging platforms, as illustrated in Figure 3.

#### 4.2.1 Requirements and methods for sample-to-sensor proximity & microfluidic device and image sensor integration

To ensure optimal performance, the microfluidic device and image sensor must be integrated in a way that maintains close proximity between the sample and image sensor. Additionally, the integration must be seamless, with the microfluidic device and image sensor properly aligned.

Close proximity between the sample and the image sensor surface serves an essential purpose for both shadow-based and luminescence-based lensless contact imaging platforms. Minimizing *z*_2_ for shadow-based imaging reduces diffraction and increases image contrast, leading to shadows that most closely resemble the sample objects. In luminescence-based lensless contact imaging platforms, minimized *z*_2_ is of particular importance since the luminescence emission is non-directional and close proximity between the sample and the pixel array maximizes photon collection efficiency by capturing the luminescence before it significantly diverges. Additionally, in a microfluidic device with multiple testing and control channels, minimizing *z*_2_ allows for accurate distinguishing of the source of luminescence emission.

**Figure 4:**
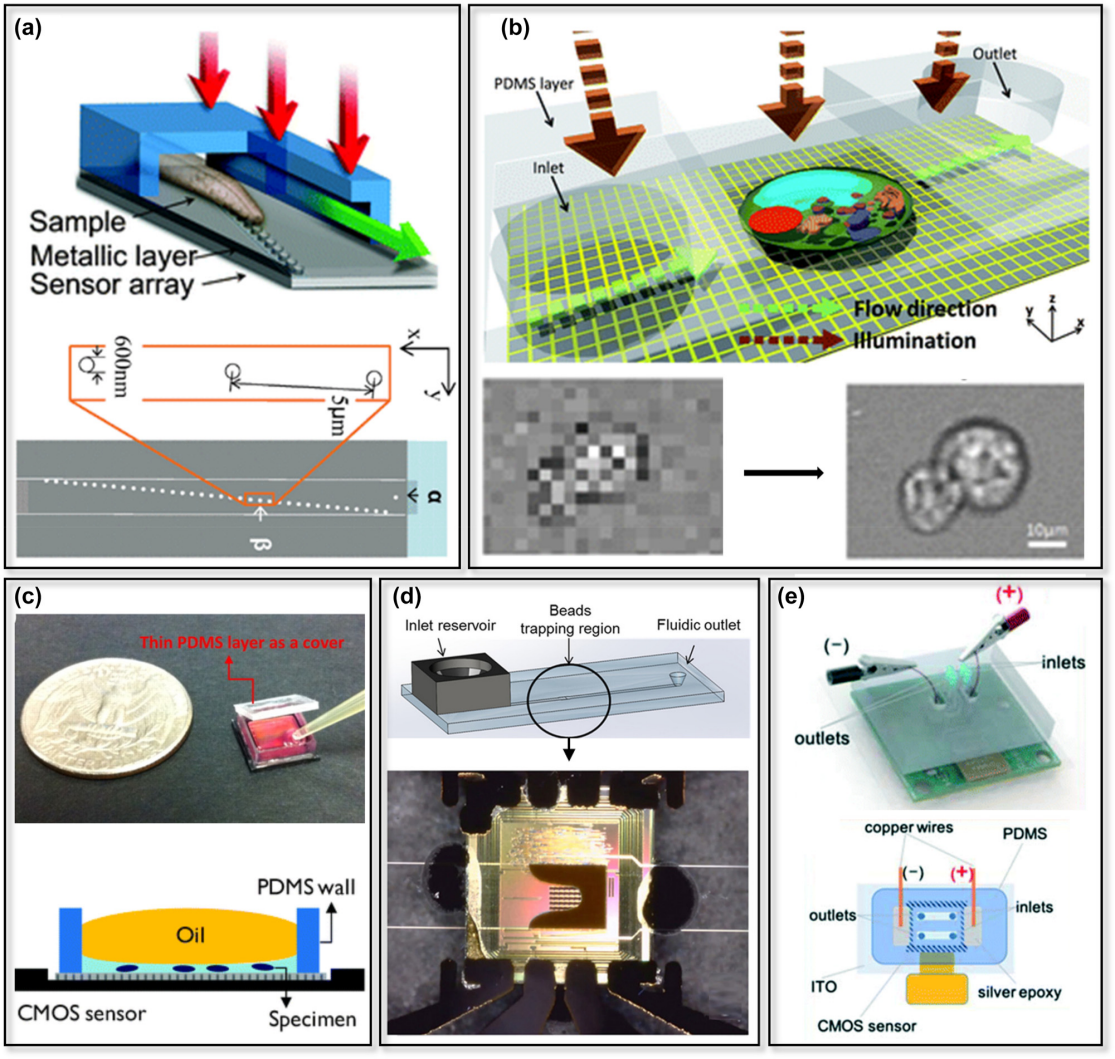
Microfluidic integrated lensless contact imaging platforms. (a) An OFM with an aluminum layer with aperture array [172]. Architecture of the OFM (top), with the red and green arrows indicating the illumination and flow direction, respectively. Top view of the OFM (bottom), with *α* and *β* denoting the isolated aperture and corresponding aperture, respectively. (b) A subpixel resolving OFM device [173]. Schematic of the device (top), low-spatial-resolution image of *Entamoeba invadens* cysts captured by the CMOS image sensor (bottom left), and the reconstructed image (bottom right) with higher spatial resolution. (c) The ePetri dish. An ePetri dish prototype with a PDMS layer as a cover (top) [174] and a schematic of an ePetri dish with an oil droplet on the sample (bottom) [175]. (d) A CL immunoassay on a microfluidic lensless contact imaging platform with magnetic beads trapping region above a CMOS image sensor [176]. (e) A microfluidic single-electrode ECL enzymatic sensor on a CMOS image sensor (top) and the top view of the device (bottom) [17]. *Modified with permission from* [17, 172–176].

To ensure close proximity, the microfluidic device is designed in a way such that the topology of the microfluidic device conforms to the image sensor chip and the microfluidic channel region above the active area of the image sensor is in direct contact with the sensor surface, as shown in Figure 4c. To achieve seamless integration, the scale mismatch between the microelectronic elements on the image sensor and the microfluidic ports must be addressed [171]. This is because the microelectronic elements on the image sensor are small in size, whereas the inlet and outlet of the microfluidic device need to be large enough, around hundreds of micrometers, for interconnection and fluid sampling. To address the issue, the entirety of the microfluidic device can be fitted on the active area of the image sensor surface, or the fluidic region can be extended beyond the image sensor chip by embedding the image sensor chip in a surrounding medium such as epoxy to obtain a planarized microfluidic channel.

#### 4.2.2 Challenges for integrating microfluidic chips onto image sensors

The merging of miniaturized microfluidics with lensless platforms is a challenging task that requires innovative solutions. The following paragraphs discuss the challenges associated with this task, along with some potential solutions that have been proposed to overcome these challenges.

Regardless of the fabrication technique, after replicating the patterns in PDMS, the most common assembly approach is to seal the channels with a solid or flexible material, including glass or silicon, or other polymers. This sealing process involves creating irreversible chemical bonds between the PDMS and substrates by activating or modifying their surfaces. Traditionally, this was often done using chemical bonding between the (–OH) groups created using oxygen plasma or chemical oxidation (Figure 2f). Given the relative ease of oxidizing the surface of silicone-based materials (glass or PDMS), they have become the most commonly used materials for sealing microchannels [177].

However, this method of bonding is mostly limited to silicone-based materials with flat surfaces which limits their application in more complex environments. For instance, when integrating with lensless platforms, bonding the microfluidic chip to the imaging sensor’s surface can be challenging, as the semi-sphere microlenses on the surface create an uneven surface (Figure 2f). To address this issue, one potential solution is to coat the chip’s surface with a thin layer (∼4 μm) of a polymer, like silicone (e.g., PDMS) or epoxy to achieve a flat surface, and then use traditional bonding techniques. This can also provide a proxy to match the refractive index of the sensor to that of the chip. However, common PDMS formulations are often too viscous to be easily spin-coated, and as such, they are often diluted with solvents like hexane, although such organic solvents also pose the risk of damaging other components of the device. Another limitation of coating the lens surface is the addition of an extra layer between the lens and the chip.

Optical crosstalk (Figure 2g) occurs when light from the same point of the sample is detected simultaneously by different pixels. This leads to artifacts and lower image quality. To address this problem, the microfluidic chip and its components need to be placed in close proximity to the photodiodes of the image sensors. Such implementation is advantageous also because it increases the photon collection efficiency. Another possible solution to optical crosstalk that is produced by isotropic (Lambertian) emitters within the sample (such as chemiluminescence and fluorescence) is to optically isolate the microfluidic components with opaque walls.

While the proximity of the sensor to the chip has its own benefits, it significantly increases the heat transfer from the sensor to the microfluidic chip (Figure 2h), thus requiring additional engineering solutions to cool the device.

Integration into peripheral equipment to load the liquid sample is another hurdle in the way of integrating lensless platforms in biomicrofluidics (Figure 2i). The primary method of propelling liquids in microchannels is using liquid pumps. Given the small volumes required in a microfluidics chip (down to picoliter), only pumps with low flow rates can be used, including peristaltic and syringe pumps. However, both of these classes of pumps suffer from pulsation and fluctuations, which directly translate to instability of the liquid in the channels [178]. One possible solution to this problem is to separate the liquid in the channels from the force being applied, using pneumatic or pressure pumps, where the liquid is pushed by the pressure of a gas, often air, behind it in the tube. This can also be applied as a vacuum force at the outlet to draw the liquid from containers at the inlet, to the channels and towards the outlet, which is connected to a vacuum source. Numerous other, less common, techniques were also explored to actively drive the liquid in the channels, including centrifugal forces [179], acoustic methods or electrokinetic flow propulsion [180]. Nonetheless, all these techniques undermine the fundamental promise of microfluidics system for being portable, easy to operate, and bringing a whole lab onto a single chip. Instead, they require a whole chest of macro-size equipment to use a micro-size chip, significantly limiting their use, especially in point-of-need settings. This limitation has resulted in a growing body of research focused on design and fabrication methods to make microfluidics systems that do not need such peripheral liquid handling equipment, using internal sources of flow control, like capillary forces.

### 4.3 Fluid control

Microfluidic lensless contact imaging platforms require careful consideration of the spatial and temporal aspects of fluid control, including delivery of the sample to the sensing area, confinement of the reaction at the sensing area, and precise timing of the reaction, to produce accurate and reproducible results.

The sample is transported by the microfluidic device towards the image sensor via capillary force, vacuum, or an actuation mechanism such as pumps or acoustic waves. Since cell counting applications using microfluidic shadow-based contact imaging require a constant flow of a relatively large sample volume, actuation mechanisms are required to automatically deliver the sample fluid. Instead of the commonly used bulky syringe pump, surface acoustic wave (SAW) actuation provides a much more compact alternative for driving the flow. To create a continuous flow in the channel, an alternating electric field is applied to a piezoelectric material, generating SAWs that create pressure to move the liquid forward [161]. For luminescence-based platforms, where a continuous sample flow through the channel is not required, sample delivery to the sensing area can be achieved by applying vacuum at the outlet of the microfluidic channel or an absorbent pad can be used with capillary force in the channel to drive the sample fluid along the channel.

Different from microfluidic shadow-based lensless contact imaging, microfluidic CL-based lensless contact imaging requires confinement of the sample at the active area of the image sensor to detect the CL emitted during reaction. Various approaches can be implemented to confine the reaction at the sensor area: microfluidic channel geometry can be used to restrict the reaction to the bottom chamber through gravity, a trapping region with physical barriers can be used to trap analyte-specific antibody-coated probe beads [176], bioprinted immobilized analyte-specific enzymes such as choline oxidase can be used to react with choline to produce hydrogen peroxide that in turn reacts with peroxidase such as HRP to produce colorimetric change [171], immobilized genotype-specific probes can be used for DNA hybridization with biotin-labeled target DNA where the hybrids are measured by CL signals produced by streptavidin-HRP conjugates that bind to the biotins [181], and magnetic trapping fields can be generated by a permanent magnet to trap magnetic probe beads [182]. Additionally, the timing of the reaction is critical, as the reaction is measured as a function of time and the sample concentration is calculated using the Michaelis–Menten equation by determining the initial reaction rate [181]. Thus, it is essential that sensing area is rapidly filled with the sample in order to accurately estimate the initial reaction rate. Though the ECL-based imaging technique serves a similar function to CL-based imaging, there are some distinct advantages. To begin with, unlike CL, ECL employs an electric potential gradient instead of reaction catalysts to facilitate the reaction, resulting in a more controlled reaction with fewer reagents required. Moreover, the electrogenerated chemical reaction only occurs on the electrode upon applying electrical potential to the electrolytic cell, providing better control over the reaction confinement and timing.

### 4.4 Experimental condition control

Temperature and pH are two of the most important experimental conditions to consider. Temperature can have a profound impact on the rate of chemical reactions and the behavior of biological samples. For example, enzyme activity and protein stability can be affected by temperature changes, and organisms may behave differently under varying temperature conditions. In the context of contact imaging on image sensors, maintaining a stable temperature is crucial, as continuous operation can cause sensors to overheat. To cool the sensor, a thermoelectric (Peltier) cooler, can be attached to the sensor board to conduct the heat generated by the sensor circuit; meanwhile, a fan and heat sink are used to effectively dissipate the heat [175, 183, 184]. Monitoring the temperature can be done by using an on-board temperature sensor [176], and a Peltier cooler and heater can work in conjunction to adjust and maintain the temperature as needed [185, 186].

When the sample is in an aqueous environment, the pH of the solution can significantly affect the activity of enzymes and the growth of microorganisms. Depending on whether the organisms are acidophiles, neutrophiles, or ornalkaliphiles, there is an optimal pH for their growth. Similarly, there is an optimal pH for the interaction of antigens and antibodies in immunoassays, as well as an ideal pH that maximizes the reaction rate by influencing enzyme activity [187]. Additionally, in luminescence-based techniques such as the luminol-H_2_O_2_ system, it is found that a pH of 10.7 provides an ideal balance between the intensity of emitted light and lifetime [17]. Maintaining an ideal pH is therefore crucial for a biomicrofluidic system. Typically, pH adjustment is done by adding acids such as hydrochloric acid and bases such as sodium hydroxide, with phosphate buffered saline being one of the most commonly used buffers [188].

### 4.5 Data processing

One of the common challenges related to point-of-care testing involves the documentation, storage, and tracking of test-related information. This information encompasses factors like experimental conditions, testing procedures, patient identification, and health indicators recorded during the test, along with the actual test results. While portable diagnostic devices can improve accessibility and availability, they often lack the rigorous quality assurance standards typically found in clinical laboratories. As a result, the importance of proper record-keeping should not be underestimated when these devices are employed in patient-care settings. The presence of portable digital storage and transmission tools like smartphones provides a potential solution to these challenges, and it’s advantageous if the testing platform can seamlessly integrate with such technologies [189, 190].

In addition to data management, computer algorithms can be used to achieve subpixel spatial resolution down to a few hundred nanometers, enabling the detection of smaller samples as well as the visualization of more defined features in the nanometer range. To achieve subpixel spatial resolution in dynamic samples, multiple frames of the sample with subpixel displacements in the channel can be recorded. Similarly, for static samples, subpixel spatial resolution can be achieved by SPSM, where the light source is shifted to generate shadows with subpixel displacements. In both techniques, the captured low-spatial-resolution (LR) images with subpixel displacements can be fused into a single high-spatial-resolution (HR) image by applying a multiframe pixel super-resolution algorithm [191–193]. As the subpixel shifting is purely translational, a simple “shift-and-add pixel” algorithm with low computational cost can be used [173, 191]. The relationship between the LR images and the HR image is mathematically represented by a function consisting of a motion vector calculated from the sample’s location in each frame, a blur operation that remains invariant in linear space for all LR images, a decimation operation based on the number of pixels in the image sensor and the desired resolution, and an additive Gaussian noise vector. A HR image is obtained by solving for the HR image in this function through a series of computations.

To avoid excessive data storage from continuous image capturing, reconstruction of an HR image from a single captured image can be achieved by single-image super resolution algorithms using machine learning, with the state-of-the-art method using convolutional neural network (CNN) [159, 194]. A CNN-based super resolution algorithm (CNNSR) is trained with pairs of LR images and corresponding ground truth HR images from a training library to recognize the correspondences between them, where the CNN is utilized to create a mapping function between the LR and HR images. To train the CNN, LR images are first scaled up using bicubic interpolation to match the size of corresponding HR images. A mapping function between LR and HR images is then learned to make the interpolated image as similar as possible to the reference HR image with minimized loss function. During the learning process, three main training layers are constructed in the CNN. In the first layer, overlapping patches are extracted from the interpolated LR image, and each patch is represented as a high-dimensional vector. The second layer uses non-linear mapping to convert the output vectors from the first layer into vectors that each represents a single HR patch. In the third layer, the previous HR patches are combined to generate one HR image that closely resembles the original HR image. This training process is performed with different LR image and HR image pairs until the network parameters produce a loss function value close to zero. Once the training is complete, the CNN can be used to generate HR images from LR images that are not part of the training library. To do this, an LR image captured by the image sensor is inputted into the trained CNN, where the LR image is resized and passed through each trained layer’s filter.

**Table:** 

**Box 2│ Key requirements and challenges for a biomicrofluidic lensless contact-based imaging platform**
Biomicrofluidic lensless contact imaging involves placing a microfluidic device directly on an image sensor, creating a portable, inexpensive, and simple platform for handling small sample volumes with high-throughput results and micrometer-level spatial resolution. To implement this platform, there are several key requirements that need to be considered. ***Effective implementation of biosensing technique*** *.* The platform employs a biosensing technique that quantifies analytes through measuring optical signals generated by reactions between the analyte and the biorecognition element such as analyte-specific enzymes or antibodies. The integrated image sensor detects the changes in colorimetry or light intensity, which can result from chemiluminescence, electrochemiluminescence, or absorbance from an LED light source during the reaction. In order for the reaction to take place and be detected by the image sensor, the active area of the sensor must be functionalized with biorecognition elements. ***Close proximity between the sample and the image sensor surface***. To achieve maximum photon collection efficiency [17], it is crucial to minimize the distance between the sample and the image sensor surface. ***Seamless integration of microfluidics and image sensor*** *.* The integration of microfluidics and image sensor components must address the scale mismatch between the miniaturized size of the image sensor and the larger size of the microfluidic input/output ports needed for interconnections and fluid sampling [171]. Additionally, the topology of the microfluidic device must conform to the image sensor to ensure proper alignment [171, 195]. ***Control and limitation of the reaction to the sensing area***. The microfluidic device must transport the sample towards the image sensor in a controlled manner via accurate fluid handling and volume control. To improve efficiency of binding events of the analytes to the bioreceptors and ensure reproducibility of the test, the reaction must be confined to the image sensor region through implementations such as microfluidic channel geometry, trapping region with physical barriers [176], bioprinted pre-immobilized enzymes [195], or magnetic trapping fields [182]. ***Regulated experimental conditions***. Physical experimental conditions such as temperature and pH must be maintained at an ideal level throughout the experiment to ensure reliable and reproducible results with minimized variation in experimental conditions. The temperature can be adjusted by thermoelectric elements, whereas the pH can be adjusted by adding acids or bases. ***Data transfer and analysis***. To analyze the readout signals from the image sensor, the recorded data must be processed by a computer program. The data is transferred from computers to FPGAs and later to ASICs for analysis. Computer algorithms such as multiframe pixel super-resolution algorithm and single-image super resolution algorithm can be employed to achieve subpixel spatial resolution of the captured images.

## 5 Methods and applications of integrated microfluidics with lensless optical image sensors

### 5.1 Shadow-based microfluidic methods

The first microfluidic lensless contact imaging system was introduced in 2005 by Lange et al., where a microfluidic culture chamber containing the sample is stacked directly on top of a CMOS image sensor and illuminated by a light source to capture images of *Caenorhabditis elegans* during spaceflight [196]. The spatial resolution was limited to approximately 10 μm by the pixel size of the image sensor at the time.

In the subsequent years, Heng et al. developed an aperture-based microfluidic lensless imaging technique with submicron spatial resolution limited by the aperture size and termed it optofluidic microscopy (OFM) [29, 172]. Constructed directly on an image sensor, the OFM comprises a PDMS microfluidic chip bonded onto an opaque metallic film with submicron aperture array etched onto its surface. As shown in Figure 4a, the aperture array is oriented at an angle relative to the microchannel, with the aperture size being several hundred nm, the aperture spacing in the *y*-direction being half the aperture size to ensure complete sampling, and the aperture spacing along the aperture array being equal to the pixel size to map each aperture onto an individual pixel. An isolated aperture *α* and corresponding aperture *β* are located at different *x*-coordinates but the same *y*-coordinate to calculate the flow velocity by dividing the distance between *α* and *β* over the time taken for the sample to travel from *α* to *β*. An image of the sample can be created using the flow velocity to un-skew the compilation of images taken during sample transmission.

Instead of relying on apertures to achieve subpixel resolution, later research in microfluidic lensless contact imaging employs computer algorithms to generate high spatial resolution images [160, 173]. As shown in Figure 4b, the sample is delivered to the image sensor by the microfluidic flow in the channel, and a sequence of images with spatial resolution limited by the pixel size is captured while the sample undergoes subpixel displacements and is then processed with a pixel super-resolution algorithm to reconstruct a single image with high spatial resolution. Besides imaging *C. elegans*, these dynamic microfluidic lensless contact imaging approaches are also used for cytometry and blood cell imaging towards disease diagnosis [160, 197].

Over the last decade, a lensless contact imaging system termed ePetri (Figure 4c) has emerged as a versatile tool for various applications, such as bacterial microcolony counting, motile microorganism monitoring, viral plaque analysis, and waterborne parasite identification [174, 175, 183, 184, 198]. Unlike other microfluidic lensless contact imaging platforms, ePetri features a static fluidic component (i.e., the culture media) for cultures of adherent cells. The ePetri platform consists of a plastic wall positioned along the edges of the CMOS image sensor surface, a thin PDMS cover or an oil droplet to prevent culture media evaporation, and an illumination source. To acquire high spatial resolution images, ePetri employs the subpixel perspective sweeping microscopy (SPSM) method, where the illumination source is sequentially tilted or shifted to produce subpixel shifting of the shadow. Compared to conventional petri dishes, ePetri dishes offer the advantage of real-time continuous imaging in a large field of view, as well as reduced labor and contamination risks.

### 5.2 Luminescence-based microfluidic methods

Compared to shadow-based microfluidic lensless contact imaging platforms, luminescence-based microfluidic lensless contact imaging platforms are relatively uncommon. Although a few CL (Figure 4d) and ECL-based (Figure 4e) microfluidic lensless contact imaging platforms have been demonstrated [17, 171, 176, 199], where light-emitting target analyte-specific reactions occur at the image sensor, the majority of luminescence-based imaging platforms rely on cameras positioned at a distance above the reaction channel to capture the light emission [200–211]. While achieving a lens-based non-contact configuration is relatively easy since it does not require close proximity between the sample and the sensor surface, it falls short in photon collection efficiency compared to the lensless contact imaging configuration, where the reaction channel is integrated with the image sensor. Therefore, the transition of luminescence-based imaging platforms into a lensless contact imaging configuration could bring significant advancements to the field of biosensing.

### 5.3 Recent applications

Microfluidic lensless non-contact imaging has been used for red blood cell classification, bacteria detection, virus sensing, particle agglutination assays for protein and virus quantification, algae monitoring for environmental condition evaluation, and bio-aerosol detection for pollen classification [212, 213]. Microfluidic lensless non-contact imaging has also been employed as polarization microscopy for imaging plant samples and birefringent samples such as synovial fluid crystals [214, 215]. Compared to these non-contact imaging counterparts, microfluidic lensless contact imaging platforms offer distinct advantages, as they are more compact, have better photon collection efficiency, and eliminate the need for image reconstruction processes. Microfluidic lensless contact imaging platforms have demonstrated versatility and strength in various applications, as summarized in Table 1, which provides an overview of their respective characteristics and performance. These platforms have been used for various cell imaging applications, including cell detection, classification, counting, and continuous monitoring [158–161, 174]. Among these applications, blood cell counting [158–160, 216] has been particularly well-performed, with the advantages of portability and automation over conventional biomedical instruments such as microscopy and flow cytometers, making it ideal for POC diagnostics. The ePetri platform has proven to be a versatile tool for continuous cell and microorganism monitoring, as well as for the identification of pathogens such as waterborne parasites and malaria [174, 175, 183, 198]. Other applications of organism imaging on biomicrofluidic lensless contact imaging platforms include the imaging of *C. elegans*, a model organism that allows for studying life from the microscale of genetics to the macroscale of environmental impact on behaviors, due to its simplicity and short generation cycle and life span [172, 196, 217]. Biosensing applications of biomicrofluidics lensless contact imaging platforms have been demonstrated using CL or ECL for the detection and measurement of metabolites, antibodies, proteins, and viruses in body fluids, as well as antibiotic residues in food [17, 171, 176, 218]. This has implications for disease diagnosis and monitoring, such as measuring uric acid concentration in saliva for gout disease and monitoring choline concentration in blood for cancer and cardiovascular disease. These sensing methods have shown comparable or superior performance to conventional sensing methods, indicating their potential to replace current diagnostic tools and their suitability for operating in POC settings. Notably, for many platforms [173, 175, 197, 217], the microfluidic channels enabled not only sensing of flowing samples but also subpixel spatial resolution by utilizing the sub-pixel displacements of the sample as they flow through the channel. Overall, the use of biomicrofluidic lensless contact imaging platforms has opened up new possibilities for live specimen imaging, POC diagnostics, and disease monitoring, demonstrating their potential to significantly impact the field of biomedical research and healthcare and beyond.

**Table 1:** Characteristics and performance of microfluidic lensless contact imaging applications.

	Author	Year	Purpose	Sample	Target	Biorecognition	Image	Pixel	Spatial	Main outcome
					analyte	element	sensor	size (μm)	resolution (μm)	
Cell imaging	Liao [216]	2019	Automated blood cell classification	White blood cells	White blood cells	–	CMOS	1.4	–	Classification accuracy of 98.44 %
	Huang [161]	2017	Automated cell detection and counting	Cells in aqueous solution	Human bone marrow stromal cells	–	CMOS	6	>6	Enhanced portability by surface acoustic wave pump; average error rate of −6.53 %
	Liu [158]	2017	Complete blood count	Whole blood	Platelet cells	–	CMOS	1.1	<2 (CNNSR)	Able to detect platelets (<2 μm); CNNSR provided 37 % increased mean structural similarity to microscope (60 X) images
	Huang [159, 160]	2014	Automated blood cell counting	Mixed red blood cell and HepG2 cell solution	Red blood cells and HepG2 cells	–	CMOS	1.1	- (CNNSR)	Percent error of 8 %; CNNSR provided 4-times higher resolution
	Zheng [174]	2011	Continuous monitoring of growing or confluent cells	Growing or confluent cells	Giemsa-stained confluent HeLa cells	–	CMOS	2.2	0.66 (SPSM)	First demonstration of ePetri dish
Organism imaging	Jung [183]	2016	Automated real-time bacterial colony counting	Bacteria culture	*S. epidermidis*	–	CMOS	2.2	0.66 (SPSM)	Minimum detectable colony diameter of 20 μm; comparable results to conventional colony-counting method
	Lee [175]	2012	Continuous monitoring of motile microorganisms	Euglena gracilis culture	Euglena gracilis	–	CMOS	2.2	0.95 (sub-pixel motion)	First demonstration of imaging motile microorganisms on ePetri dish
	Zheng [173]	2010	Imaging unicellular organisms	Unicellular Protists in aqueous solution	Euglena gracilis and *Entamoeba invadens* cysts	–	CMOS	3.2	0.75 (sub-pixel motion)	First demonstration of algorithm-based subpixel resolving lensless contact imaging
	Pang [217]	2010	Color imaging of nematodes	*C. elegans*	*C. elegans*	–	CMOS	3.2	0.8 (aperture based)	Capable of color imaging
	Heng [172]	2006	Nematode imaging	*C. elegans*	*C. elegans*	–	CCD	5	0.49 ± 0.04 (aperture based)	First demonstration of aperture-based subpixel resolving lensless contact imaging
	Lange [196]	2005	Studying behavioral effects of spaceflight on nematodes	*C. elegans*	*C. elegans*	–	CMOS	10	>10	First demonstration of lensless contact imaging
Pathogen identification	Lee [198]	2014	Waterborne parasite identification	Stool	Giardia, Cryptosporidium, and Entamoeba cysts	–	CMOS	2.2	<1 (SPSM)	Identification accuracy: Manual ≥ 98 % Automatic ≥ 96 %
	Lee [197]	2011	Blood cell imaging for malaria diagnosis	Whole blood	P. Falciparum -infected red blood cells	–	CMOS	2.2	0.66 (sub-pixel motion)	Manual identification accuracy of 88 %
Biosensing	Abbasi [17]	2022	Metabolite quantification	Saliva or urine	Uric acid	Uricase	CMOS	1.4	>1.4	First demonstration of ECL on lensless imaging platform LoD: 26.09 μM; 8-fold photon collection efficiency than other ECL imaging platforms
	Annese [171]	2022	Metabolite quantification	Blood plasma	Choline	Choline oxidase	CMOS	100	>100	LoD: 3.2 μM
	Cetin [219]	2021	Virus detection	Nasal swab	H1N1 viruses	H1N1 virus antibody	CMOS	–	–	LoD: 10^3^ TCID_50_/mL
	Kao [218]	2018	Antibiotic quantification	Food	Ciprofloxacin	*E. coli*	CCD	–	–	LoD in milk, egg white, and chicken essence: 8 ng/mL; LoD in egg yolk: 64 ng/mL
	Van Dorst [176]	2016	Antibody, protein, and peptide quantification	Blood plasma	Immunoglobulin G (IgG) Amyloid ß42 Inducible protein 10 (IP-10) John Cunningham virus Polyomavirus capsid protein 1 (JCV VP1) specific antibody	Protein A Amyloid ß42 specific antibody IP-10 specific antibody Anti-human IgG	CMOS	–	–	Antibody LoD: 7-Fold lower than traditional colorimetric plate-based ELISA Protein LoD: 200 fM Peptide LoD: 460 fM

CNNSR, convolutional neural network based super-resolution; SPSM, subpixel perspective sweeping microscopy; LoD, limit of detection.

## 6 Conclusions and outlook

Analyzing complex liquid samples requires specialized equipment that is typically only available in centralized laboratories, presenting a challenge as these devices often require large volumes of samples that are not always available. The field of biomicrofluidics has evolved to address this constraint by the development of capillaric components that can be integrated into complex microfluidic devices designed for specific use cases, leading to increased availability of liquid sensing methodologies for applications at the point of care or point of need. However, one challenge remains, as the signal of interest needs to be detected and quantified. In conventional devices, this is usually performed by external optical or optoelectronic systems that record the photons from the sample, perform analysis of the signal, and convert it to an electrical signal that can be further amplified and digitally processed. Typically, these devices are microscopes or similar systems that operate based on similar principles. However, due to their size and lack of portability, their use at the point of need is limited. To address this problem, complete integration of the microfluidic system with the detection system is needed. While many solutions have been proposed previously, in this article we discuss one of the most promising approaches, which is the use of lensless semiconductor image sensors for the contact-mode integration of the optoelectronic and microfluidic components.

As described throughout the article, different modalities of contact-mode lensless on chip imaging have been demonstrated, such as transmission, dark field, and luminescence, with spatial resolution limited only by the pixel size. In addition, CMOS architecture and manufacturing technology of integrated photodiodes with readout electronics allow for low power requirements, low electronic noise, and high quantum yield for conversion of photons into electrons, while the combination with microlens arrays allows for high photon collection efficiency, with millions of micrometer- or sub micrometer-sized pixels being arranged in a square unit cell pattern in a centimeter-sized chip to provide portability, high spatial resolution, and large FOV. Given the above advantages, it seems logical that this imaging approach is currently being explored for integration with microfluidic components and devices towards the goal of integrating sample handling, bioreceptor distribution, transducer, and reader into a single compact, inexpensive, and user-friendly device.

There are, however, significant challenges that need to be addressed for successful integration. First, the sample needs to be in close proximity (<10 μm) to the surface of the image sensor. This requires the microfluidic chip and its components to be attached to the image sensor chip, which is not trivial but can be achieved through chemical or physical treatment of the two interfaces. Adequate barriers for microfluidic components such as channels and chambers also need to be prepared to ensure proper sample handling. Second, functionalization of the sensor surface needs to be performed while maintaining the integrity and functionality of the device. Mild chemical treatment and surface functionalization with a bioreceptor need to be carefully chosen and applied to the specific regions of the device (channels, chambers) that are designed for analyte binding. Third, experimental conditions such as fluid flow, pH and temperature, need to be carefully controlled given that the image chip generates a significant amount of heat during operation. Finally, data collection and analysis need to be performed, where dedicated ASICs can be developed to address the issues of cost and portability.

In addition to the previously mentioned engineering challenges, there are fundamental improvements that may lead to broader use and wide-spread adoption of microfluidic lensless contact imaging systems. First, current image sensors provide limited spatial resolution, as determined by the Nyquist criterion for spatial sampling rate, where the spatial resolution is approximately twice the pixel size. To overcome this limitation, reducing the pixel size will improve the spatial resolution, enabling imaging and sensing of smaller objects and allowing for increased capabilities not only for mechanistic and functional studies but also for higher levels of multiplexing and high throughput. In addition to reducing the size of individual pixels in the image sensor, spatial resolution can be improved by multi-frame subpixel resolving techniques such as combining sub-pixel shifted images into an image with subpixel spatial resolution, as well as single-frame subpixel resolving techniques using machine learning methods such as CNN to generate an image with subpixel spatial resolution from a single input image. Second, the implementation of fluorescence in contact-mode lensless imaging systems is yet to be fully realized, as challenges exist in removing excitation light from the fluorescence signal. If this challenge can be addressed, implementations of fluorescence-based biosensors will become possible. Third, reducing the size of microfluidic components will be necessary to match the dimensions of the semiconductor image sensor and provide opportunities for multiplexing, which comes with fabrication and liquid handling challenges that will need to be addressed.

Overall, the integration of microfluidic devices and components with semiconductor optical image sensors provides new opportunities for diagnostic at the point-of-need, with broader availability for testing, whether it is for medical applications such as liquid biopsies or nonmedical applications such as food safety, environmental monitoring, and pathogen detection. Specific examples include the detection of disease biomarkers related to cancer, neurodegenerative, cardiovascular, or infectious diseases, as well as testing for heavy metals, endocrine disruptors, plant pathogens or other environmental pollutants in remote areas that do not have access to testing equipment. In addition, the development of 2D detector arrays with sub-micrometer-sized pixels and sub-pixel resolution imaging techniques will open up further opportunities for the analysis of nanometer-sized particles such as small extracellular vesicles that play a significant role in physiological and pathological processes. Current technologies lack capabilities for comprehensive morphological and chemical characterization of these particles, thus the advancement towards nanometer-level lensless on chip imaging could lead to a better understanding of these particles and their roles in diseases.

Finally, most point-of-need biosensors such as glucose sensors, pregnancy tests, and rapid infectious disease sensors are lateral flow assay devices where the liquid is moving through a membrane by capillary action towards a test and control line that are functionalized for a specific analyte. However, these devices have significant limitations when it comes to sensitivity and multiplexing ability. These limitations can potentially be addressed by the methodologies discussed in this article, offering new opportunities for improved diagnostic capabilities at the point of need.
